# An exploration of changes in the mental models of middle management and their association with activities to implement a dialogue tool to address mental health in the workplace

**DOI:** 10.1186/s12889-025-21702-x

**Published:** 2025-02-14

**Authors:** Lily Joanne Anzion, Frederieke G. Schaafsma, Cécile R. L. Boot, Margriet A. G. Formanoy, Roosmarijn M. C. Schelvis

**Affiliations:** 1 Department of Public Health, Amsterdam UMC, Amsterdam Public Health Research Institute, University of Amsterdam, Academic Medical Center, Amsterdam, The Netherlands; 2 Netherlands Institute of Mental Health and Addiction, Utrecht, The Netherlands

**Keywords:** Organisational-level mental health intervention, Participatory Action Research, Middle-management, Mental models, Group-level sensemaking

## Abstract

**Background:**

There is a growing awareness for addressing mental health in the workplace. Although interventions to promote mental health at the organizational-level exist, implementation is a challenge. Middle management can play a crucial role in implementing organisational-level interventions. Also, we know that mental models often need to change first, before enactment of such interventions occur. The aim of this study is therefore to better understand whether and how changes in mental models of middle managers are associated with the implementation activities of an organisational-level intervention to support mental health. Ultimately, this is meant to lead to the enactment of, in this case, a dialogue tool to normalize a conversation between middle manager and employee to enhance mental health.

**Methods:**

Participatory Action Research was used as a method to design our implementation of a dialogue tool primarily focused on middle management to address mental in health in one SME company with 238 employees. In-depth interviews with 11 (middle) managers at the start of the implementation phase were held. After nine months, 9 out of the same 11 (middle) managers were interviewed again to gain understanding whether and how changes occurred during the implementation. Thematic analysis was applied to the interviews, from inductive and deductive perspective. Focus groups with employees and observations during the implementation activities were used for triangulation purposes.

**Results:**

The mental models of the (middle) managers were analyzed based on readiness for change, perceived challenges and perceived opportunities. These mental models were generally positive towards the project, despite the low trust towards the Top Management and the general lack of experience with addressing mental health at the workplace. Nine months later, mental models changed towards more awareness and engagement in addressing mental health. Also, enactment of the dialogue tool by middle management and employees occurred. An association of these changes with, for example, the frequency and pace of implementation activities in which all employees were involved was reported.

**Conclusion:**

To implement interventions addressing mental health at the workplace, taking the time and using implementation activities that match the needs of middle management might help to change mental models of (middle) management.

**Supplementary Information:**

The online version contains supplementary material available at 10.1186/s12889-025-21702-x.

## Background

There is a growing awareness for addressing mental health in the workplace [1, 2]. It is estimated that 15% of the adults of working-age deal with mental health issues. Knowing that more than half of the world’s population is working, this is a significant number [3]. Employers are confronted with the effects in the workplace by, for instance, absenteeism, presenteeism, reduced productivity and increased staff turnover [1, 4]. Employers are willing to address these challenges, but are often not well equipped to act accordingly [2]. A growing number of organisational-level interventions that address mental health exist [1]. However, the implementation of those interventions remains a challenge [5, 6]. Because of this, only few interventions truly change organisational reality due to, for example, a lack of employee participation and support [1, 5, 7, 8].

Also from an academic perspective, awareness is raised to better understand mental health at the organisational level [6]. Burdorf (2023) highlights that there is still a lack of insight into the pathway of effectiveness of the organisational-level health interventions [9]. He states that it is important to not only evaluate the effectiveness of interventions, but also take into account the specifics of the organisational context and implementation process. For this we need a more comprehensive approach than the traditional Randomized Controlled Trial that is focused on standardizing the context. This may also ask for a different outcome measure. For example, not the number of sickness absences, but a measure for organisational changes that acknowledges the constant interaction of individual behavior and working conditions [7, 9–11].

To highlight the organisational context and actors in research on mental health in the workplace the focus could be more on the role of middle management. Previous research has shown that middle managers in the workplace play a crucial role in *driving the change* in an organization in general [10, 12]. More specific to the theme of mental health, middle managers are essential for identifying signals of employees that struggle with mental health and acting accordingly [3, 7, 13].

In this study specifically, we focused on the implementation process of a dialogue tool that addresses mental health in a simple and comfortable manner by normalizing a conversation about this issue, between employees and their managers [14]. The *Traffic Light System* offered a basic framework, but required co-creation in order to embed it at the organisational-level. Participatory Action Research (PAR) design is an established method for co-creation as it offers the opportunity to transform the context in which the study is taking place [15]. This is done by not only engaging the study population, but letting them be part of the change [7, 16]. By using PAR as a study design focused on the middle manager, we also address the call of Nielsen et al. to include participation and the role of middle management during all phases of the implementation [12, 17].

In the context of implementation, we know that for true organisational changes to occur, managers and employees first need to unlearn their old mental models, in order to learn new ones [18, 19]. Mental models are a person’s reference that is used to make sense of the world [20] and ultimately steer our behaviors and influence the level of ‘theory-in-use’. Theories-in-use are not only the attitudes, values, policies and practices that are verbalized, but also enacted [21]. When implementing an intervention, we aim for enacted behavior. Therefore, in this study, we refer to ‘enactment’ when discussing the change in behavior with the application of the *Traffic Light System* at the organisational-level.

If we want to understand implementing mental health interventions that lead to enactment, we need to zoom into the more intangible phenomena of changing mental models during these processes. This study aims to better understand if and how changes in mental models of middle managers are associated with the implementation activities of a mental health intervention (i.e. a dialogue tool), that is meant to lead to the enactment of new behavior that supports mental vitality in the workplace.

## Method

### Setting

The study took place at a plastics manufacturing company, a Dutch SME located in the Eastern part of the Netherlands. The company has 238 employees, divided over two factories that work in shifts.

### Intervention

The *Traffic Light System* is a dialogue tool that evokes to answer the question “How am I doing?” in different domains (work, private life and personal life) and aims to prevent stress related complaints [14]. Private life refers to the interactions you have with family and friends, whereas personal life refers to the things you do for yourself (e.g. hobby). By using the colors green, yellow and red to rate how you are doing, a common language is created for employee and managers. Yellow and red refer to the degree of severity of perceived stressors. The tool also highlights existing and potential energy sources that correspond with the color green. Overall, it helps with reflecting and speaking about prevention, guidance for return to work, but also the sustenance of healthy employees. In a pilot study, the tool showed a potential positive effect on stress prevention and related sick leave [22].

While initially focused on the Top Management Team and (middle) managers (*N* = 27), the intervention was ultimately rolled out for all of the remaining 211 out of 238 (91,2%) employees. In this study, the participants received a self-management book “Stress te lijf met energie” that explains the *Traffic Light System* [14]. Also, the study population received a four hour training held in April 2023. Subsequently, two practice sessions of both two hours were held in June and October 2023, in which “Traffic Light System” conversations were practiced using role plays. Moreover, co-creation was done, in which the study population’s perspective on embedding the tool at an organisational level was asked. The detailed process is elaborated in the next section.

### Design

The design of the study was a Participatory Action Research (PAR) consisting of five cycles, with four phases each: (1) designing (2), executing (3), reflecting (4), documenting and sharing. A combination of qualitative methods was used to collect and analyse data in these phases (i.e. interviews, focus groups, observations and logbooks).

PAR is based on the idea that knowledge is co-produced and results are co-created by academics, as well as the stakeholders and research population itself [12, 17]. By co-creating with the population that is ultimately going to enact the intervention, ownership and sustainability of the changes can be increased, possibly leading to practical and social impact [16]. This is in line with other studies in occupational contexts [23, 24] and in community contexts [25, 26] that aim to let their population be a part of social change.

### PAR project team

The PAR project team consisted of four researchers from the Amsterdam University Medical Center with expertise in (mental) health in the workplace and organisational change management, the author of the “Traffic Light System”, a researcher from the Trimbos Institute, and the HR director, a Continuous Improvement engineer, the operational manager, and an employee representative from the company involved. This selection of people was based on a combination of academic experts and a representation of employees of the company. This mix enabled the Participatory Action Research to be executed from both external academic- and internal, practical perspectives.

### PAR cycles

This project was designed as a process of *five cycles* ((0) preparation; (1) assessing needs; (2) intervention training; (3) intervention development through co-creation; (4) intervention embedding), with *four phases* each ((1) designing (2), executing (3), reflecting (4), documenting and sharing [27]). Also, a member-check was done after each cycle to make sure that members were given the space to reflect and hence co-create the next cycle. Table 1 describes activities in each cycle, and how member-checks were executed.

**Table 1:** Description of the PAR cycles, phases, activities and how member check was executed

Cycle	Phase	Description of data collection and implementation activities	Member check
**0. Preparation**	Designing	The PAR-project team was composed and the PAR design was made for the grant application.	The main stakeholders were consulted for the grant application of the study.
	Executing	The PAR-project team kicked off the project with a meeting.	
	Reflecting	In the kick-off meeting, the PAR-project team reflected on what needed to be done in the first cycle.	
	Documenting and sharing	Through messages on the screens in the two locations of the factory the start of the project was shared with all employees, and names and a photo of the researchers were shared.	
**1. Assessing needs**	Designing	• The design and planning for this cycle started by the PAR-project team finding participants who voluntarily wanted to join the interviews and focus groups to assess needs concerning mental health in the workplace.	The interviews were cross-checked by the participants by sending them a summary of the interview.
	Executing	• 11 in-depth interviews were held by the researchers with the study population (top and middle managers) on the current status of mental health in the organization and their needs concerning mental health in the workplace. • A kick-off meeting was organized with the study population (middle and Top- Management) and a delegation of the PAR-project team. • Two focus groups with a total of 20 employees were conducted to capture also employees’ perspective on needs for mental health interventions in the workplace.	
	Reflecting	A three hour reflection session was held with the PAR-project team, to reflect on the outcomes of this phase.	
	Documenting and sharing	• Observations were done and documented in a logbook during the activities (i.e. interviews, kick-off meeting, focus groups) by the primary researcher (author LA). • The outcome of the interviews and focus groups was shared on screens in the factories, together with contact information of the PAR-project team for additional questions or concerns. • Info sessions were held by the Top- and middle managers in their teams to kick-off the project, inform employees on the various stages of the project and what would be expected of them, and to answer questions.	
**2. Intervention training**	Designing	• The content of a training as a preparation for using a dialogue tool, was based on the needs of the Top- and middle management and the needs of their employees.	On a voluntarily basis, nine of the participants of the training were contacted by phone to retrieve their feedback on the training and understand their needs as input for further cycles in the process.
	Executing	• The training ‘preparation for using a dialogue tool’ was given to the top and middle management by an experienced occupational physician who developed a dialogue tool (and who was also part of the PAR-project team).	
	Reflecting	A three hour reflection session on the training and other activities thus far was held with the PAR-project team.	
	Documenting and sharing	Observations were done and documented in a logbook during the activities (by author LA). Results from this cycle were shared on the screens in the company.	
**3. Intervention development through co-creation**	Designing	Based on the needs of Top- and middle management, a practice- and design session for adjusting a dialogue tool to the factory context and needs was designed by a part of the PAR-project team.	• Info sessions were organized to present a project up-date as well as to receive feedback from all employees. • After careful consideration, the self management book about the Traffic Light System was made available to all employees too.
	Executing	• Top- and middle management received a self-management book with description and examples of a dialogue tool, called ‘Traffic Light System’ as preparation for the practice session. • Top- and middle management participated in a practice session with the dialogue method. The dialogue method was practiced twice in the form of a role-play with various cases. • A design session was held with the middle managers and employees, in order to inventory needs for further development and embedding of the intervention in the factory.	
	Reflecting	A three hour reflection session was held with the PAR-project team to reflect on the design and practice sessions and the project thus far.	
	Documenting and sharing	Observations were done and documented in a logbook during the activities (by author LA). Results from this cycle were shared on the screens in the company.	
**4. Intervention embedding**	Designing	• Based on the feedback from the prior cycles, the PAR project team designed an approach for other SME's, creating the concrete deliverables and learnings from the PAR. • Also, the researchers designed a second interview guide for the aim of better understanding the deeper layers of the study population’s experience and possible changes.	• The interviews were cross-checked by the participants by sending them a summary of the interview. • Also the opportunity was given to share their feedback and experience on the entire process.
	Executing	• 9 interviews with Top- and middle managers were held to understand if and how mental models changed during the successive cycle stages. • A second round of focus groups with employees was done to enhance the understanding of the collective mental model. • A roadmap of types of embedding the intervention in other SME-contexts was designed. • Lessons learned are put into a guide for HR, managers and the Employee Representative Council, as well as in an infographic.	
	Reflecting	A three hour reflection session was held with the project team.	
	Documenting and sharing	Observations were done and documented in a logbook during the activities (by author LA). Results from this cycle were shared on the screens in the company.	

Abbreviation. SME: small to medium enterprise

### Participants

The study population consisted of *N* = 27 participants and represented the complete Top Management (*N* = 6) and all (middle) managers (*N* = 21) of the company. During the project’s kick-off session, the study population was asked to voluntarily participate in interviews. These participants were all male with an age range of 25–63 years. In the first round (February 2023), 11 participants were interviewed for this study. In the second round (October 2023), 9 out of these same participants were interviewed again. Due to sick leave and retirement, two participants could not be interviewed again. Informed consent was obtained from all participants before the start of the interviews.

### Data collection

We used data triangulation by combining three different methods: interviews, focus groups, observations and logbooks. The focus groups, observations and logbooks were used to complement and interpret the results from the interviews.

#### Interviews

Two rounds of in-depth interviews were held. In the first cycle, in February 2023, 11 semi-structured, in-depth interviews were held with 9 middle managers and two members of the Top Management. The aim of the initial interviews was to assess perceptions and appraisals on the current status of mental health in the organisation and needs of the participants regarding this topic. The interviews were held in Dutch by two researchers (authors LA and RS). Interviews took 30–60 min and were located in the HR office of the company. The interviews were audio recorded and transcribed. A summary of the transcript was shared with the participant for corrections. The interview guide was based on the needs assessment as a part of the first PAR cycle. The laddering technique was used to elicit underlying perspectives and emotions in the context of mental health at work [28]. Other cognitive techniques were used, such as Think-Aloud, with which participants share what comes to mind with regard to a certain topic [29]. This was followed by scripted probing questions, to enhance standardization of the interviews [29]. Examples of questions are: “How do you experience the company’s culture in the context of mental health?” and “What opportunities and challenges do you perceive if you consider implementing the Traffic Light dialogue tool in your team?”, the complete interview guide can be found in supplementary file 1.

A second round of interviews was done with the same nine participants in cycle 4, in October 2023, with seven middle managers and two members of the Top Management. Interviews were held to understand if and how the representation of the mental model changed during the PAR cycles. The central open question here was: “Did you experience changes since the Traffic Light project started?”. The second question was: “Can you explain when and how these changes occurred?”. Participants based their answers regarding the perceived changes on recalling the past. When it was hard for participants to recall their initial perceptions, we used data from their first interview as a method to probe.

#### Focus groups and observation log for triangulation

To validate the perspective of the (middle) management in the interviews, we used triangulation based on focus groups with employees and an observation log. In cycle 1 and 3, focus groups were held with 20 out of the 238 employees who were not part of the (Top or middle) management team. Focus group participants were asked to voluntarily participate. These employees were selected, taking into account the representation of both factories and office workers, with the large majority being factory workers. Informed consent was obtained from all participants before the start of the focus groups.

Throughout all of the cycles, observations of the focus groups, the project meetings and co-creation sessions, field notes of the on-site visits in the factories were documented in a logbook. The first author (LA) was present at all main and most minor events to document planned and unplanned processes. The logbook also helped to enhance understanding of researcher reflexivity.

### Data analysis

#### Thematic analysis

This study used thematic analysis to analyse the data that came from the different sources [24, 30]. The interviews, that were held in the first cycle, were coded in an inductive way with open codes using the program MaxQDA. Two authors (LA and RS) cross-checked the codes, to enhance the robustness of the codes. After inductively coding the first round of interviews, they were deductively coded using the suggested themes in the systematic review of Havermans et al. [31] on process evaluation of stress management interventions in the workplace, i.e.: readiness for change, perceived challenges and opportunities for a mental health intervention in the organisation. We gained insight in the representation of the mental model of the Top- and middle management regarding current mental health status and how to address this in their workplace. Given what had happened during the action oriented PAR cycles, we wanted to explore if, and if so what had changed in this mental model representation. Therefore, the second round of interviews were again inductively coded to find if changes in mental models occurred and how they are associated with the implementation activities. The criteria for reporting qualitative research (COREQ) were kept in mind [32]. We used the individual level interview data to do an analysis of themes at the group level, validated by the data of the focus groups and the logbook.

## Results

The results are structured as follows. First, the representation of the collective mental model of the participants before implementation of the dialogue tool is described based on the inductive and deductive analysis of interviews in PAR-cycle 1 (assessing needs) (Table 2). Second, any changes in representation of mental models and enactment of the dialogue tool that occurred during PAR cycles 2–4 are described (Table 3). Third, the perceived association of occurred changes in the mental models with the implementation activities during PAR cycles 2–4 organized by the PAR-project team is explained (Table 4). Overall there was no obvious difference between the mental model of the Top Management members and middle managers. However, the theme: ‘Example behavior of the Top Management’ was obviously only reported by middle managers during the second round of interviews. In this result section we refer to participants, unless there is an obvious difference in the perspective of the middle manager and Top Management.

### Mental modle of (middle) management before the implementation phase

Before implementation, the following themes were found that represented the mental models of (middle) management.

**Table 2:** Mental model of the (middle) management before implementation

Main themes^2^	Sub-themes^1^	Quote
Readiness for change	Low trust towards the Top Management and organisational change fatigue	*“Like I said*,* with some people in the past… some things happened which led to losing their trust. That is quite hard to repair. In that case*,* it will be an obstacle for a person to speak about it openly.”* (Interview 2)
	Overall positive attitude towards the project	*“I think it is a great idea. Does not matter for which company or sector. We have to take care of the boys and receive the signals*,* if they’re there. And sometimes… there are no signals*,* because they lock everything in. And if they come to you saying ‘it’s too much’*,* yes*,* than you need to do something. ”* (Interview 5)
Perceived challenges in the context of a mental health intervention	Mental health stigma	*“Male culture. Yes*,* men together. We laugh a lot. Jokes are being made. But… not many people talk about their situations at home. Mostly work related stuff is talked about.”* (Interview 9)
	Existing differences in approaches of middle managers in addressing mental health	*“Now a lot is dependent on if the supervisor gives space and whether he picks up the signals.”* (Interview 3)
	Lack of experience in addressing mental health	*“If I look at the situation with a lady that’s in my team*,* she apparently has an issue with telling me she is dealing with an issue. I thought it was okay*,* but apparently it’s not. I understand that you’re not super open about these things*,* especially mental health. Nobody likes that. If you don’t feel well*,* you will not toot your own torn.”* (Interview 6)
Perceived opportunities in the context of a mental health intervention	Willingness to learn new skills and conversation techniques	*“Maybe there is a guideline*,* that would be easy. How you deal with something like that. For personally*,* I don’t think I need it*,* but I can imagine sometimes there is a need for something that guides you or that gives you agenda points that you can think about.”* (Interview 5)
	Seeing possibilities to embed the intervention at the organisational-level	*“You could say that 75% of our employees need to be green in one of the domains. That could be a target.”* (Interview 7)

Note. ^1^The sub themes emerged from the inductive coding. ^2^ The sub themes were deductively coded and clustered into main themes

### Readiness for change

#### Low trust towards top Managementand organisational change fatigue

Before implementation, low trust towards the Top Management was experienced as a result of several events. For example, the acquisition of the organization by an American Holding, changed many of the procedures and ways of working. Participants therefore mentioned that they were tired of organisational changes. They also spoke about a lack of communication from the Top Management about these organisational changes, which they reported to cause mistrust.

#### Overall positive attitude towards the project

In general, all participants thought addressing mental health was relevant, while still being skeptical about the success of implementation.

### Perceived challenges in the context of a mental health intervention

#### Mental health stigma

All participants described their organisational culture as emotionally closed. They explained that this is partly determined by the local culture of the region, but also by the stereotyped male culture. Most participants reported that they, for example, often discuss what they did over the weekend. However, discussing more private matters, such as mental health, was rarely done. The majority mentioned that the stigma around mental health was perceived as a challenge for this project to succeed.

#### Existing differences in approaches of middle managers in addressing mental health

The majority elaborated on the existence of differences in the personalities and leadership qualities. Some of the participants already developed their personal ways of dealing with mental health in their team, while others reported to have no experience at all. This was perceived as a challenge, because of the possible mismatch of the middle managers’ baseline knowledge on mental health with the intervention’s training.

#### Lack of experience in addressing mental health

Also, multiple participants mentioned that the lack of experience in addressing mental health could be a challenge. They explained that it might be hard for them to identify mental health issues for themselves and their employees. Also, they reported not to have the tools to probe when it is clear that someone is not doing well, but is not openly speaking about their situation. This was seen as a challenge that the project team took into account.

### Perceived opportunities in the context of a mental health intervention

The majority of the participants perceived opportunities in putting mental health on the agenda.

#### Willingness to learn new skills and conversation techniques

Depending on the existing experience with addressing mental health in their team, most of the participants were open to more knowledge and skills in this context. Especially the opportunity for learning about conversation techniques was perceived.

#### Seeing possibilities to embed the intervention at the organisational-level

The majority of the participants saw the opportunity for embedding the dialogue tool in the organisation’s procedures. They reported that they were willing to make the dialogue tool part of the annual performance review or other recurring meetings. Others saw the opportunity to embed the dialogue tool in the Top Management’s strategy and targets.

### Changes in mental models and enactment during implementation

Nine months later, all participants reported changes in their mental models, as well as enacted behavior changes. They are described below.

**Table 3:** Changes in mental models and enactment during implementation

Main themes	Sub-themes	Quotes
Changes in mental models – intangible changes	More awareness for the theme of mental health	*“But*,* it’s on my mind. I also notice that in myself*,* you are more aware and you look at the boys in a different way*,* and maybe you ask different questions. You’re more aware of the whole mental health theme than before.”* (Interview 8)
	Sustained positive attitude, but a larger support base at the employee-level	*“I think that when something is a general term*,* then it will be referred to more easily when it is really needed. It needs to be generally known and accepted. I think that has been achieved.”* (Interview 6)
	Enhanced openness towards each other	*“I notice that the boys are being more open. I already had more boys coming to me when something was up. That is good. That makes it easier for us. If you don’t get certain signals*,* than you are not able to capture- and act on them.”* (Interview 5) *“If I look at the last six months*,* how many people shared something that they would not have done before… I think that something changed there.”* (Interview 6)
Enactment of the Traffic Light tool – tangible changes	Language changes in informal conversations and shift reports	*“You see it around. Sometimes in a report. Something like: my Traffic Light was yellow or red. It now is colloquialism. I think that’s good. That already is revenue.”* (Interview 4) *“Such a metaphor with Traffic Lights*,* everybody gets that. It was not like that before. […] Now you can easily say: I feel green*,* yellow or red.”* (Interview 8)
	Embeddedness of the intervention in formal meetings	*“I use it for the conversations I have with my employees. […] I now made it structurally […] and in those meetings I use it*,* as an agenda point.”* (Interview 6)

### Changes in the mental models – intangible changes

#### More awareness for the theme of mental health

The majority of the participants reported a change in the way they thought about addressing mental health in their organisation. They were more aware of the relevance of speaking about how they were doing in their conversations with colleagues.

#### Sustained positive attitude and larger support base at the employee-level

Before implementation, the majority of the participants reported a positive attitude towards the intervention itself, while being negative towards the expected success of the implementation. During implementation, however, the participants themselves became more positive towards the implementation process and also reported that their employees were positively receiving the Traffic Light Tool. The substantial communication that was done by the project team to update employees was reported to be of positive impact.

#### Enhanced openness about mental health towards each other

All participants reported that they experienced more openness about mental health towards each other. More than before this project started, people shared how they were doing using the *Traffic Light* colors, even if it was about private life. The ‘unknown’ of opening up, about mental health or life events in general, gradually decreased during the implementation process. Given the closed and stereotyped male culture, the majority of the participants reported that it was new to them that colleagues opened up in this way.

### Enactment of the Traffic Light tool – tangible changes

#### Language changes in informal conversations and shift reports

All participants reported that the language of the ‘red’, ‘yellow’ and ‘green’ colors, associated with the Traffic Light, was used to indicate how they were doing or how their day had been. The colors were used in informal conversation, in shift reports and sometimes as a joke. It was also mentioned that the simplicity of the Traffic Light helped in using it.

#### Embeddedness of the intervention in formal meetings

The Traffic Light was also introduced as a part of formal meetings, such as in the general performance reviews but also as the start of Top Management-, middle management- and team meetings. Also, the *Traffic Light System* was embedded in their digital HR system, as a way to request and report dialogues.

### Association of the changes in mental models and enactment with the implementation activities

The following themes are based on the implementation activities that were organized with the PAR project team and were reported to be associated with the changes in mental models and enactment of the dialogue tool.

**Table 4:** Association of the changes in mental models and enactment with the implementation activities

Main themes	Quotes
Attention given to mental health during the implementation	*“It’s just attention. I think that this whole project helped in that. Attention towards the individual and private life.”* (Interview 6)
Involvement of all employees in different settings	*“I think that something is repeated multiple times and that is it not something owned by a small selective group. […] But that everybody has hear of it. That everybody could share their input. It is being addressed in different angles. Focus groups*,* presentations for middle management*,* presentations for everybody… not betting on one horse.”* (Interview 5)
Top Management’s commitment and exemplary behavior	*“You notice that the MT*,* but also HR*,* that they are emphasizing that this is very important. That this is something that in the past was not such as thing*,* but that more and more young people are burned out and that we really need to address that.”* (Interview 5)
Frequency and pace of the implementation activities	*“I think that power of repetition caused people to use it.”* (Interview 4) *“The entire design of the whole process. The start and how we rolled in. Not very direct… […]*,* like slowly rubbing it in. That there was sufficient time in between the sessions. Not that it was every week*,* or so. So it could also be absorbed slowly.”* (Interview 5)
Foreseen challenges for the continuity of enacting the dialogue tool in the future	*“We have to continue this*,* that is the most important. Keeping it high on the agenda. Not that*,* if you leave in a while*,* we just let it go all at once. No*,* I believe in it*,* continue this and make it a part of your daily work.”* (Interview 1) *“It is often that*,* […]*,* if external companies will inform people […]*,* than it will be ongoing. […] because now there was this peek and in reality it is easily forgotten. That is not what we intent to. So if it can be repeated as a certain base*,* than you keep the attention and ultimately it will grow.”* (Interview 8)

#### Attention given to mental health during the implementation

A remarkable finding is that all participants described different forms of ‘attention’ given to the project by the project team and the Top Management, as a way to explain why changes in their mental models and enacted behavior occurred. This is attention to mental health as a topic in general, but also towards the employees as individuals, who are more than just part of the workforce.

#### Top Management’s commitment and exemplary behavior

Another type of impactful attention that was reported by the majority of the (middle) managers, was the commitment and exemplary behavior of the Top Management. The Top Management emphasized the importance of mental health awareness and also opened up by sharing their own Traffic Light status in group meetings.

#### Involvement of all employees in different settings

All of the participants mentioned that involving all employees during the implementation was an important factor explaining why change occurred. Different aspects of involvement were mentioned: the involvement of different departments and teams of the organization, different types of activities for involving them (e.g. co-creative poster sessions, presentations, and role plays) and the thorough project communication. A pitfall that was mentioned by two of the middle managers is the risk of over-asking them in terms of time and input for extra projects. When being over-asked, genuine engagement can drop.

#### Frequency and pace of the implementation activities

Both the frequency and the pace of the activities of the implementation process were reported to be associated with the occurrence of change. The power of repetition was mentioned by some of the participants. Also, the importance of the build-up of the activities was reported to be of impact.

#### Foreseen challenges for continuity of enacting the dialogue tool in the future

The majority of the participants mentioned that they would foresee that the ‘attention’ for the topic needs to remain for the intervention to be continually enacted.

## Discussion

The aim of the study was to better understand whether changes in mental models of middle managers occurred during this PAR study and if so, how these changes were associated with the implementation activities of a dialogue tool to address mental health at the workplace. Results indeed showed that, after nine months, changes in the mental models of managers did occur over time. Also, we found different types of enactment of the dialogue tool, meaning that the intervention was not only introduced but also applied. That changes in mental models lead to enacted behavior is in line with previous research [8, 10, 18].

However, over the course of the study, we zoomed in on how this change process unfolded. The activities that were reported to be of influence on these changes, seemed strongly related to the principles of Participatory Action Research [16]. Important examples of working principles were the way attention was given to the theme of mental health by involving all the employees in different settings. Also, the build-up and repetition of activities were reported as a cause of the changes. This is in line with the PAR principle that highlights the importance of constant feedback and engagement of the study population [16].

A possible underlying mechanism of the associations found, could be the phenomenon of group-level sensemaking. This is when individual mental models merge to join in a collective process of understanding new reality [33]. When looking at the common denominator of the implementation activities of this PAR, we find that it is mostly about making sense of the theme of mental health together. It refers to the process in which organisational members interpret situations in and via interactions with others, both informal or formal, in the form of discussions, negotiations, gossip, stories, and rumors [33]. Multiple sensemaking studies discuss the influence of social processes by, for example, explaining how social interaction impacts organisational change outcomes [33, 34]. It could be that activities that facilitated group-level sensemaking, in line with the PAR principles, are linked to changes in mental models [35].

Also, it is worth mentioning that the changes occurred over time, despite the initial stigma in this company. The stigma on mental health is not unique for this company only, but is found in many workplaces [36]. Managers are not always equipped, nor comfortable with the theme of mental health [37]. This is highlighted by Brouwers [38] recent research on self- and social stigma around mental health in the workplace. This stigma causes speaking about mental health in the workplace to be extra challenging [39]. This study, however, shows how implementation activities that facilitated group-level sensemaking reduced this stigma. To illustrate, managers reported that the group activities, such as the middle management trainings and role plays in which also Top Management Team members joined, led to a reduction of the ‘unknown’ and therefore decreased stigma on mental health. The implementation activities gave them the opportunity to gradually make sense of the theme together and slowly open themselves up about it. Another probable explanation for stigma reduction is the exemplary behavior of both the TopManagement Team as the middle managers. When they started to share how they were doing, more employees may have felt the space to do this as well. This is in line with Kampen (2019) who report that managers should be in the lead in order for new behavior to become normalized [40]

Therefore, *how* an organisational-level intervention to address mental health is implemented seems to be of impact. This is in line with the recently published systematic review of Genrich et al. (2022) that also highlights the relevance of focusing on managers’ mental models and perceived organizational norms towards a mental health intervention, in order for implementations of such organizational-level interventions to succeed [10]. It is than probably not so much about practically implementing an intervention (e.g. training the managers or introducing an e-learning), but more about organizing time and space at multiple organisational levels for mental models to gradually change, stigma to slowly reduce and ultimately new behavior to be enacted that enhances mental health in the workplace. This more subtle approach seems to promising in improving and addressing the complexity of implementations. Despite the focus on SME’s, these insights and overall recommended approach are also applicable to larger organizations. In an updated version of the Traffic Light System approach that is not part of this study, an online environment is developed, which simplifies scaling up and monitoring the process and outcomes of using the Traffic Light System.

### Methodological considerations

Some methodological issues require consideration. The underlying mechanisms that were found are, although in line with other studies, limited to this case-study. It could also be said that the starting point of the project contained low hanging fruit. There was no existing program at the workplace already addressing mental health, so any given attention to the theme was a gain. However, this could also be seen as challenge, as employees experienced stigma concerning the theme. Also, the study was limited to a year. A longitudinal study that evaluates changes over several years is needed for more robust conclusions. A strength is our PAR approach, that resulted in deeper human connections made with the study population [16]. This could have led to more openness and truthful findings, but may also have caused socially accepted answers during the interviews.

### Implications for future research and practice

We recommend that future research focuses on better understanding how mental models of middle managers can change, in the context of organisational-level interventions addressing mental health in the workplace, and what specific group-level sensemaking spaces are related to this. In this way, it could be easier to identify the working principles for stigma reduction and successful implementations of mental health interventions. A similar study design could be undertaken in different settings, with different organisational contexts, to increase reliability of the findings. An advice for employers who wish to address mental health at the workplace is to focus more on how to change mental models of employees. Employers should spend extra time and effort on understanding their employees’ mental models in the context of mental health in the workplace. Only then can they facilitate aligned group-level sensemaking activities that support gradually making sense of the theme together. This may lead to stigma reduction and ultimately to new enacted behavior that supports mental health in the workplace. The PAR principles, such as bottom-up needs assessments and involving all employees in co-creation sessions, serves as an inspiration [16].

Lastly, research could also investigate whether barriers exist at the employer level that might hamper even the consideration of implementing a dialogue tool and how to overcome these barriers. Examples of barriers might be stigma, or other influences of local cultural context, especially in low to middle income countries.

## Conclusion

Focusing on changing mental models of (middle) management can be an important lens for more successful implementations of organisational-level interventions that aim to enhance mental health in the workplace. *How* to change mental models and enacted behavior of employees in this context could be done by taking the time for implementation activities that hold space for group-level sensemaking and match the needs of (middle) managers. This seems to be a promising approach to improve the implementation of organisational-level mental health interventions.

## Electronic supplementary material

Below is the link to the electronic supplementary material.

Supplementary Material 1

## Data Availability

The data used and analyzed during the current study are available from the corresponding author on request.

## Abbreviations

SME: Small and Medium Enterprise
PAR: Participatory Action Research
