# Supplementary material for: An exploration of changes in the mental models of middle management and their association with activities to implement a dialogue tool to address mental health in the workplace
Source: BMC Public Health. 2025 Feb 14;25:623. doi: 10.1186/s12889-025-21702-x (PMC11829359; doi:10.1186/s12889-025-21702-x)
Supplement: Supplementary file 1 — Supplementary Material 1 [file 12889_2025_21702_MOESM1_ESM.docx]

# Supplementary file 1

# Questions semi-structured in-depth interview PAR cycle 1 *original version in Dutch

**Introduction**

Thank you for participating in this interview. The aim of this interview is to gain insight into the obstacles and opportunities for acting upon mental health at [organization]. This interview will take approximately 1 hour. We will record the interview and use this recording for data processing and analysis. The data will be anonymized and therefore cannot be traced back to you as a person. You have already given permission for this.

**Getting to know each other/making a report**

- Can you tell us what role you have within [organization] and what your job duties are?
- Do you have any questions or comments before we continue the interview?

**Introductory presentation**

How did the presentation come across to you? Have you thought about the project and/or the method – how do you look at this now?

**General experience of mental health at [organization]**

- General experience of the culture (questions about the general attitude towards mental health)
  - How do you experience the general attitude of [organization] colleagues when it comes to mental health?
  - Is the topic discussed? What kind of conversations are these? How open is the culture?
- What do you understand by mental health?
  - How do you experience mental health within [organization]?
- Absenteeism (ask about the actual situation)
  - How do you experience absenteeism within [organization]? (e.g. a lot or a little, long-term or short-term absence)

**Current approach to mental health (at the moment)**

- What is currently being done about mental health at [organization]?
- What is your experience with this approach?
- Do you have an approach yourself to deal with the mental health of colleagues?
  - If so, what is it?
  - How do you experience it? What works and what doesn't?
  - Where do you see the dividing line between the responsibility of the person themselves, the manager and the organization?
- We noticed that a lot was shared about the experience with the MT in the area of ​​changes in recent years.
  - Do you recognize that?
  - Do you think that is an obstacle to the approach to mental health? If so, who should do something about it?
- Do you ever talk to your colleagues about mental health?
- If yes, how does this work?
- If no, why not?

**Needs approach mental health (desired situation)**

- What do you need when it comes to an appropriate approach for mental health?
- If you were to sketch the ideal situation; what would it look like?
- What kind of moments are there for conversations/meetings? For example, handover, day start? Method to which we can link the Traffic Light System?
- What is needed in your role?
- What is needed from the organization/HR?
  - What is needed in the field of training for managers?
  - To what extent is the role of HR important in contact with the employee?
  - Should HR talk to the employee more often? Would that be useful?
- What are the preconditions for a conversation between employee and manager?
- Where do you see obstacles or challenges in the field of mental health at [organization]?
- Where do you see opportunities in the field of mental health at [organization]?

**We have heard that many projects are starting at [organization]. What is needed to complete this project?**

- Example: which other processes (Employer Branding, safety, performance management, recruitment and selection, management development) can this method connect to?
  - Confidential advisor: how can we give them more of a role?
  - Self-reflection as a challenge and condition for entering into the conversation. How do people view this?

**Conclusion**

- Do you have any other things that come to mind in line with what we have discussed that you would like to share?
- Do you have any other things that come to mind in line with what we have discussed that are important or useful to know for the action research?
- What next? You will receive a summary with the main points of the interview. You can read this to confirm whether you agree with it.

# Interview guide PAR cycle 4

*original version in Dutch

**Research questions we keep in mind**

*1. Do middle managers report changes?*

*2. How are (changes in) mental models associated with the implementation process?*

**Introduction**

Great that you are participating in an interview again. We will record it, as previously included in the consent form.

The reason for this interview is that we are nearing the end of the Stoplight project. Two important components that we will deliver are a scientific article and an infographic and working methods for other SMEs. So we are mainly looking for the lessons that we can learn from this project. For this we need your unvarnished opinion. So please share with us as transparently as possible what comes to mind about how you have experienced the project so far.

**Interview questions**

- Has anything changed since the Stoplight System project started?
  - If yes, what?
    For example: do you use the Stoplight System, is mental health discussed differently, have the conversations around 'how things are going' changed, etc.
  - If not, why not?
- Looking back at the process, were there specific moments that changed something?
- Probing the implementation process – showing the timeline and activities.
  - Do you recognize the following examples of participatory approach components?
  - Have these influenced your attitude towards mental health and the Traffic Light System?
    1. Is there anything that stands out/strikes you?
    2. Is there anything that you have found particularly useful?
    3. What is being talked about a lot as a result of the project?
    4. What could we have left out?

**Conclusion**

First of all, thank you very much for your participation. We will send you a summary so that you can add any corrections.

- Is there anything else you would like to share that has not been discussed?
- Do you have any questions?

# Timeline and activities

**December 2022** Kick-off with the project team

**February 2023** In-depth interviews with Roosmarijn and Lily

**March 2023** Two focus groups with employees

**April 2023** Middle management training by Peter Ribbens

**June 2023** Middle management session practicing cases and embedding co-design session and focus groups with employees

**September 2023** Information sessions from [organization] about project status and coherence with other projects around mental vitality

**October 2023** Middle management session, deepening from Peter Ribbens and practicing cases

**Other informal activities**Informal conversations with each other about the project 'in the corridors'

Physical information "Stress te tegen met energie." book
